# Safinamide in the treatment pathway of Parkinson’s Disease: a European Delphi Consensus

**DOI:** 10.1038/s41531-022-00277-z

**Published:** 2022-02-21

**Authors:** Fabrizio Stocchi, Angelo Antonini, Daniela Berg, Bruno Bergmans, Wolfgang Jost, Regina Katzenschlager, Jaime Kulisevsky, Per Odin, Francesc Valldeoriola, K. Ray Chaudhuri

**Affiliations:** 1 Department of Neurology, University and IRCCS San Raffaele Pisana, Rome, Italy; 2 Parkinson and Movement Disorders Unit, Center for Rare Neurological Diseases (ERN-RND), Tübingen, Germany; 3 Department of Neuroscience University of Padua, Padua, Italy; 4 Department of Neurology, UKSH, Campus Kiel, Christian-Albrechts-University Kiel, Kiel, Germany; 5 Department of Neurodegeneration, Hertie-Institute of Clinical Brain Research Tübingen, Tübingen, Germany; 6 Department of Neurology, AZ St-Jan Brugge-Oostende AV, Campus Brugge, Bruges, Belgium; 7 Department of Neurology, Ghent University Hospital, Ghent, Belgium; 8 Parkinson-Klinik Ortenau, Wolfach, Germany; 9 Department of Neurology and Karl Landsteiner Institute for Neuroimmunological and Neurodegenerative Disorders Klinik Donaustadt, Vienna, Austria; 10 Movement Disorders Unit, Neurology Department, Hospital de la Santa Creu i Sant Pau, Barcelona, Spain; 11 Department of Medicine, Autonomous University of Barcelona, Barcelona, Spain; 12 Centro de Investigación en Red sobre Enfermedades Neurodegenerativas (CIBERNED), Madrid, Spain; 13 Division of Neurology, Dept of Clinical Sciences Lund, Lund University, Lund, Sweden; 14 Universitat de Barcelona Institute of Neurosciences, 537114 Barcelona, Catalunya Spain; 15 Institut d’Investigacions Biomèdiques August Pi i Sunyer, 146245 Barcelona, Catalunya Spain; 16 Centro de Investigación Biomédica en Red sobre Enfermedades Neurodegenerativas, Barcelona, Catalunya Spain; 17 Hospital Clinic de Barcelona, 16493, Movement Disorders Unit, Neurology Service, Barcelona, Catalunya Spain; 18 Parkinson Foundation Centre of Excellence, King’s College Hospital and Kings College, London, UK

**Keywords:** Parkinson's disease, Parkinson's disease

## Abstract

Safinamide is a highly selective, reversible MAO B-inhibitor recently marketed in European and North American countries. To better define clinical indications regarding motor and non-motor symptoms, targeted population and safety of this compound, ten movement disorders specialists, experts in their field, convened and developed a panel of statements on: the role of glutamate in Parkinson’s disease, introduction to fluctuations, efficacy of safinamide on motor symptoms, motor complications and non-motor symptoms, quality of life, safety of safinamide and target population for use. Strong consensus was reached for all the statements on the efficacy of safinamide on motor symptoms, motor fluctuations, quality of life and safety. Among non-motor symptoms, a positive consensus was reached for the symptoms sleep/fatigue, mood, and pain while there was a lack of consensus for the statements regarding the efficacy of safinamide in improving cognition, urinary and sexual functions. The statement on orthostatic hypotension obtained a negative consensus. The consistent and large agreement reached in this Delphi panel perfectly reflects the perception of efficacy, safety and tolerability of safinamide as evident from pivotal trials and clinical practice and shows how these findings may guide movement disorders specialists in their clinical therapeutic approach. The impact of non-motor symptoms in PD is considerable, and management remains an unmet need. In this context, the ability of safinamide to impact some non-motor symptoms may represent the most promising and distinctive feature of this compound and deserves further investigations.

## Introduction

Monoamine oxidase B inhibitors represent an important treatment option in the management of Parkinson’s disease (PD), both in the early and in the advanced stages of motor complications^1,2^. The clinical benefit of MAO-B inhibitors arises from the ability of these medications to enhance the level of dopamine by decreasing its catabolism in the brain.^3^. Among MAO-B inhibitors, safinamide has a dual-mechanism of action since it is able to inhibit (a) MAO-B, potentiating dopaminergic transmission and (b) glutamate release by blocking voltage-dependent sodium channels and modulating calcium channels^4,5^. Safinamide is a benzylamine derivative which acts as potent, highly selective, and reversible MAO-B inhibitor. Due to its reversibility^4,6^, treatment with safinamide is associated with reduced risk of hypertensive crises or serotonergic syndrome^7,8^ as well as drug interaction^4,6^.

Safinamide is marketed in Europe and in North America under the brand name of Xadago® (Zambon Pharma) and Onstryv® (Valeo Pharma); it was approved in 2015 as adjunctive therapy to levodopa in mid- to late-stage fluctuating patients and became commercially available in the spring of 2016. While its efficacy in controlling motor symptoms and improving motor fluctuations is well established^9–11^, uncertainty remains on its potential ability to control dyskinesias in the long term or to address the many non-motor symptoms that are typical of the advanced stages of the disease. The aim of this study was to obtain a European Consensus on the use of safinamide, considering the efficacy of this compound on motor symptoms and motor complications, its effect on non-motor symptoms (NMS), quality of life in patient with PD and how its clinical effect is perceived by clinicians. Moreover, we wished to identify an ideal target population and delineate the safety in different PD patient sub-populations.

## Results

Hundred and nineteen panelists among movement disorders specialists were identified in the following countries: Italy, UK, Belgium, Spain, Germany, Sweden, Austria, Netherlands. The response rate was 76% (*n* = 90) which was considered high, taking into account that the study was performed during the COVID 19 pandemic and related lockdown in most countries.

Strong consensus was reached for all the statements regarding the efficacy of safinamide on motor symptoms, motor fluctuations, quality of life and safety (29/34 statements, 85,2 % of agreement, mean score 91.5), suggesting a shared view of European movement disorders specialists on these topics. Among NMS, a common, positive consensus was achieved for the symptoms sleep/fatigue, mood, and pain while there was a lack of consensus for the statements regarding the efficacy of safinamide in improving cognition, urinary and sexual functions. No agreement was reached either on the tolerability of safinamide in patients with hallucinations. The statement on orthostatic hypotension obtained a negative consensus.

A second round of questionnaire was deemed unnecessary by the Board, since all the statements with no or negative agreement were considered clearly stated and results obtained reflected trends in clinical practice.

Table 1 summarizes the statements and the percentage of agreement/disagreement reached, based on the responses of the 90 Panelists.

**Table 1:** Consensus score.

		Disagreement (score 1-2)	Agreement (score 3-4-5)
TOPIC 1 - Glutamate pathway role in Parkinson’s Disease			
1.1	Glutamate is involved in the pathophysiology and pathogenesis of PD.	4%	96%
1.2	Glutamate overactivity observed in PD basal ganglia contributes to the occurrence of motor symptoms such as hypokinesia, bradykinesia and rigidity	20%	80%
1.3	Glutamatergic neurotransmission in the basal ganglia is relevant for the normal control of movement	4%	96%
1.4	Glutamatergic neurotransmission in the basal ganglia is relevant for the normal control of pain, cognition and mood	7%	93%
1.5	Abnormal firing across the glutamatergic corticostriatal pathway has gained support as a key mechanism contributing to dyskinesia	2%	98%
1.6	Abnormalities in cortical glutamate levels may play a role in decline of executive functions in early PD and in the development of dementia in advanced PD.	14%	86%
1.7	An increase in glutamate signaling can play a role in inflammatory pain	20%	80%
1.8	An increase in glutamate signaling can play a role in neuropathic pain.	7%	93%
TOPIC 2 - Introduction to fluctuations			
2.1	Wearing OFF can be present in patients taking three doses of levodopa daily	1%	99%
2.2	An early fluctuator is a patient who has had motor fluctuation for no more than one year	23%	77%
2.3	The use of a questionnaire (such as WOQ19, WOQ9) is useful in the diagnosis of WO	7%	93%
TOPIC 3 - Efficacy of Safinamide: Motor Symptom			
3.1	Safinamide is not just an MAOB inhibitor	2%	98%
3.2	Safinamide improves motor symptoms (UPDRSIII) in the short and in the long term	2%	98%
3.3	Safinamide reduces OFF time in patients with fluctuation (motor complication)	0%	100%
TOPIC 3 - Efficacy of Safinamide: Motor Complications			
3.4	The Glutamate-modulating component of safinamide may contribute to its clinical effects of increasing “on” time without troublesome dyskinesia.	6%	94%
TOPIC 3 - Efficacy of Safinamide: Non Motor Symptom			
3.5	Safinamide improves orthostatic hypotension	72%	28%
3.6	Safinamide improves sleep/fatigue	20%	80%
3.7	Safinamide improves mood	17%	83%
3.8	Safinamide improves cognition	40%	60%
3.9	Safinamide improves urinary function	46%	54%
3.10	Safinamide improves sexual function	60%	40%
3.11	Safinamide is effective for the management of pain in PD	19%	81%
TOPIC 4 - Quality of life			
4.1	Safinamide improves QOL in PD patients	2%	98%
TOPIC 5 - Safety of Safinamide			
5.1	Safinamide is a safe add-on therapy for symptomatic PD treatment.	0%	100%
5.2	Safinamide increases “on” time without increasing troublesome dyskinesia.	4%	96%
5.3	Safinamide 100 mg improves dyskinesia in the long term	27%	73%
5.4	Safinamide is well tolerated in patients with cognitive impairment	11%	89%
5.5	Safinamide is well tolerated in patients with hallucinations	39%	61%
5.6	Safinamide should be dosed as 100 mg daily within 2–4 weeks if 50 mg daily is tolerated well	14%	86%
5.7	Safinamide as an adjunct therapy in patients aged ≥75 years with advanced PD is safe and tolerated	5%	95%
5.8	The reversible effect of MAOB inhibition like safinamide can be an advantage in clinical practice	1%	99%
TOPIC 6 - Target Population			
6.1	Safinamide is an effective and safe add-on to levodopa therapy in PD	0%	100%
6.2	Safinamide is a valid therapeutic option in early stages of fluctuations	3%	97%
6.3	Safinamide is a valid therapeutic option in patients with advanced PD	2%	98%

### Topic 1: Glutamate role in Parkinson’s disease

**Fig. 1:**
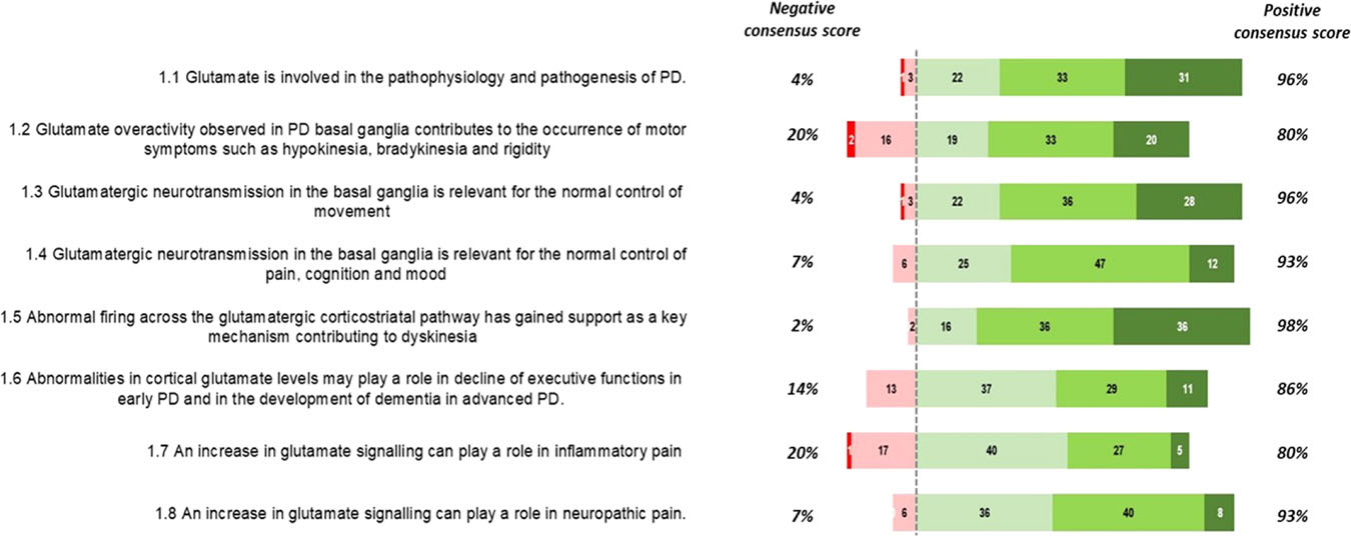
Delphi questionnaire results: topic 1 “Glutamate pathway role in Parkinson’s Disease”. Numbers in the colored bars are the total number of votes received for each level of disagreement/agreement (1, extremely disagree; 2, disagree; 3, agree; 4, mostly agree; and 5, extremely agree). The “negative consensus score” and the “positive consensus score3 are percentage.

Statements 1.1–1.8 (Fig. 1): the glutamatergic circuits within the basal ganglia were confirmed to have a relevant role in the normal control of movement (96% agreement), pain, mood, and cognition (93%). A large consensus was reached on the statement that the glutamatergic pathway is involved in the aetiopathogenesis of the disease (96%) and that the abnormal firing across the glutamatergic cortico-striatal pathway is a key mechanism in the development of dyskinesia (98%). Panelists´ opinions on the contributing role of glutamate in the emergence of motor symptoms of PD and in executive dysfunctions were less convergent, even if consensus was obtained for both statements (80 and 86%). Glutamate was considered to be involved in neuropathic pain (93%) but to be less crucial in the development of inflammatory pain (80%).

These statements reflect the high level of scientific evidence which has confirmed and defined the role of glutamate in motor and non-motor symptoms of PD^12,13^.

### Topic 2: Introduction on fluctuations

**Fig. 2:**
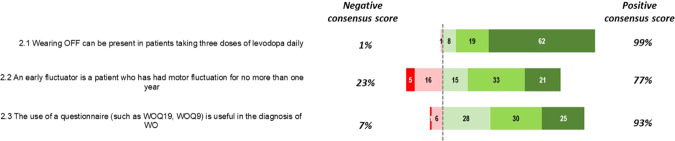
Delphi questionnaire results: topic 2 “Introduction to fluctuations”. Numbers in the colored bars are the total number of votes received for each level of disagreement/agreement (1, extremely disagree; 2, disagree; 3, agree; 4, mostly agree; and 5, extremely agree). The “negative consensus score” and the “positive consensus score3 are percentage.

Statements 2.1–2.3 (Fig. 2): the Panelists agreed that an early fluctuator can be defined as a patient who had motor fluctuations for no more than one year and that wearing off phenomena can be present in subjects taking no more than three doses of LD a day (99%). Ninety three percent of the Panelists found the use of WOQ19 and WOQ9 to be useful in the diagnosis of wearing off, in agreement with existing literature^14,15^.

### Topic 3: Efficacy - Motor symptoms

**Fig. 3:**
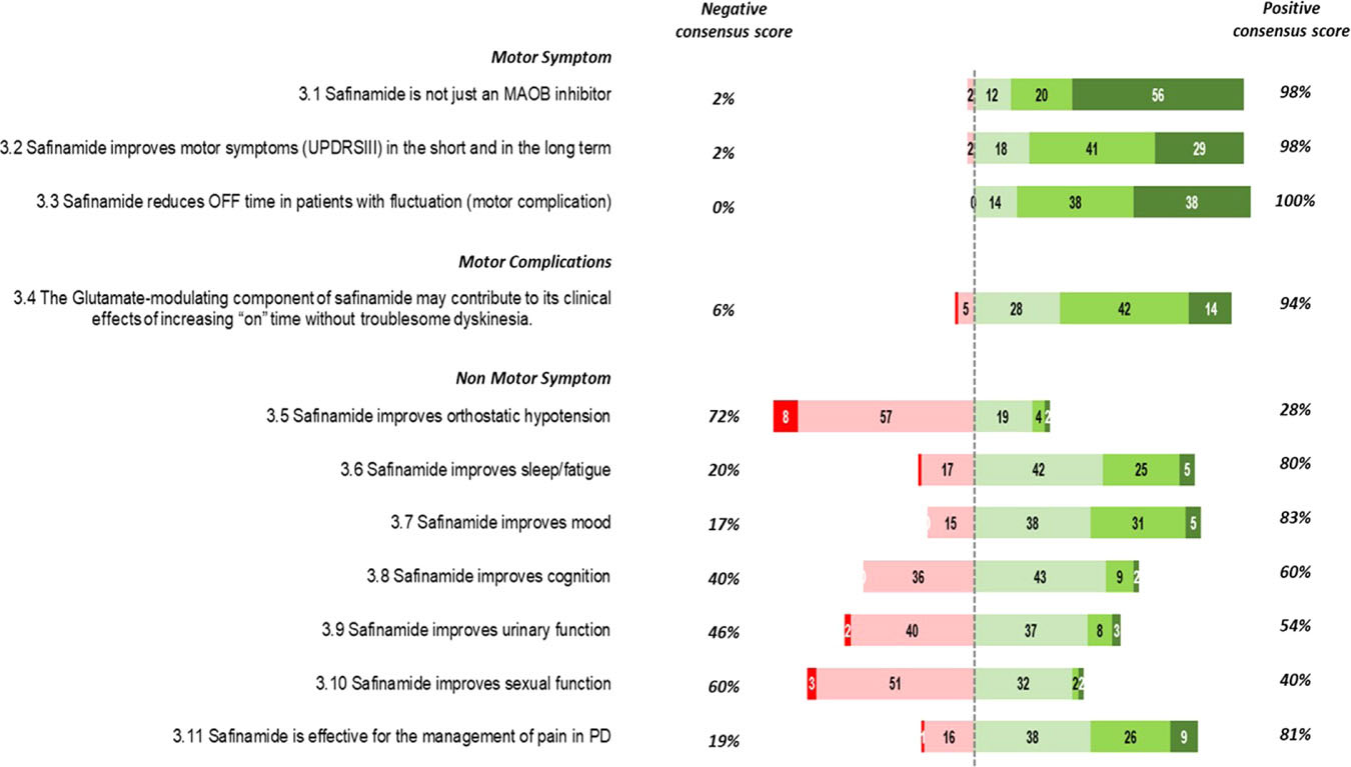
Delphi questionnaire results: topic 3 “Efficacy of Safinamide”. Numbers in the colored bars are the total number of votes received for each level of disagreement/agreement (1, extremely disagree; 2, disagree; 3, agree; 4, mostly agree; and 5, extremely agree). The “negative consensus score” and the “positive consensus score3 are percentage.

Statement 3.2 (Fig. 3): there was almost unanimous consensus on the ability of safinamide to improve motor symptoms, both in the short and long term (98%).

### Topic 3: Efficacy - Motor fluctuations

Statements 3.1 and 3.3–3.4 (Fig. 3): There was full agreement on the efficacy of safinamide in reducing off time (100%). The efficacy of safinamide was believed to be related not only to MAO-B inhibition (98%) and that the glutamate-modulating component of this compound contributes to its clinical efficacy in increasing good on time (without troublesome dyskinesia, 94%).

### Topic 3: Efficacy - NMS

Statements 3.5–3.11 (Fig. 3): a positive consensus was achieved for the symptoms sleep/fatigue, mood, and pain (respectively 80, 83 and 81%) while no agreement was observed in relation to cognition, urinary and sexual function. Seventy-two percent of the Panelists disagreed with the statement: safinamide improves orthostatic hypotension.

### Topic 4: Quality of life

**Fig. 4:**
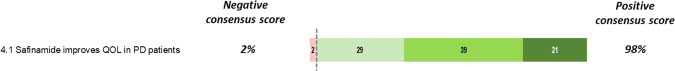
Delphi questionnaire results: topic 4 “Quality of life”. Numbers in the colored bars are the total number of votes received for each level of disagreement/agreement (1, extremely disagree; 2, disagree; 3, agree; 4, mostly agree; and 5, extremely agree). The “negative consensus score” and the “positive consensus score3 are percentage.

Statement 4.1 (Fig. 4): The positive effect of safinamide on quality of life reached an almost unanimous consensus (98%).

### Topic 5: Safety

**Fig. 5:**
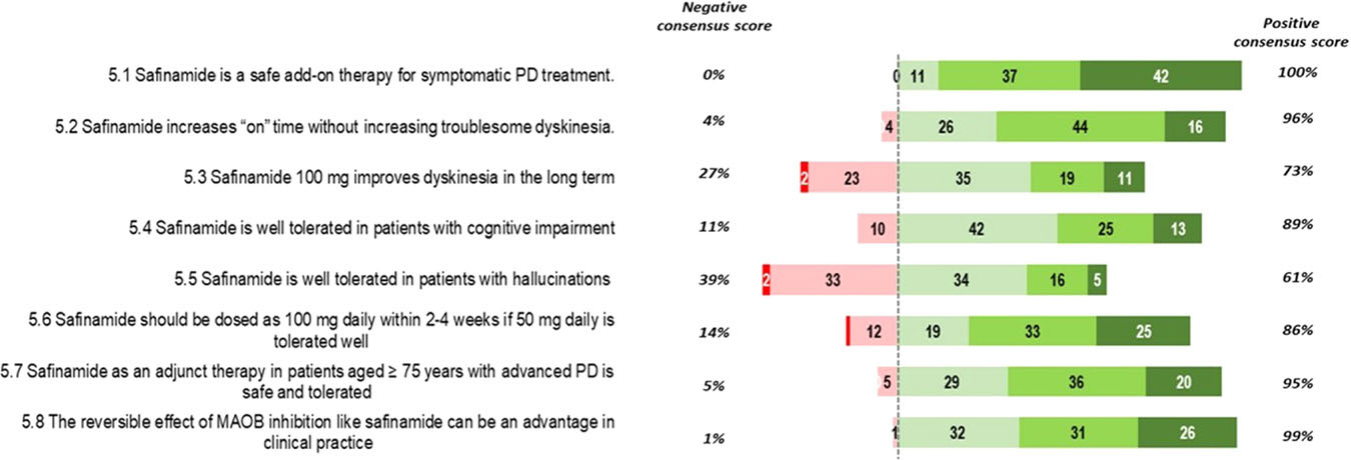
Delphi questionnaire results: topic 5 “Safety of Safinamide”. Numbers in the colored bars are the total number of votes received for each level of disagreement/agreement (1, extremely disagree; 2, disagree; 3, agree; 4, mostly agree; and 5, extremely agree). The “negative consensus score” and the “positive consensus score3 are percentage.

Statements 5.1–5.8 (Fig. 5): the totality of the Panelists agreed with the statement that safinamide is safe as add-on therapy for symptomatic PD treatment and that its reversible inhibitory effect of MAO-B represents an advantage in clinical practice. Ninety-six percent of voters believed that safinamide could ameliorate on time without increasing troublesome dyskinesia but only 73% were convinced that the molecule is able to improve dyskinesia in the long-term. Agreement was reached regarding the safety of safinamide as an adjunct therapy in patients aged ≥75 years (95%) and in patients with cognitive impairment (89%). No consensus was reached regarding the tolerability of safinamide in patients with hallucinations (39 vs 61%).

### Topic 5: Safety - Dyskinesia

Statement 5.3 (Fig. 5): The long-term efficacy of safinamide on dyskinesia reached a relatively weak agreement (73%), reflecting the conflicting results available from the literature to date.

### Topic 6- Target population

**Fig. 6:**
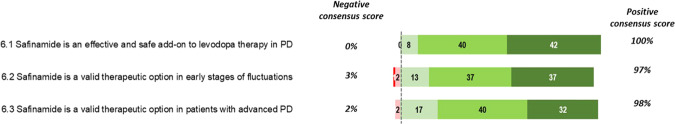
Delphi questionnaire results: topic 6 “Target Population”. Numbers in the colored bars are the total number of votes received for each level of disagreement/agreement (1, extremely disagree; 2, disagree; 3, agree; 4, mostly agree; and 5, extremely agree). The “negative consensus score” and the “positive consensus score3 are percentage.

Statements 6.1–6.3 (Fig. 6): safinamide was considered a valid therapeutic option in patients with advanced PD (100% agreement), in the early stages of motor fluctuations (97%) and as add-on to LD therapy in PD (100%).

## Discussion

The results of this Delphi survey suggest that safinamide is a useful treatment option for PD patients suffering from motor fluctuations and some specific non-motor fluctuations. The broad beneficial effect of this compound can most likely be ascribed to its multimodal mechanism of action, which is dopaminergic (reversible MAO-B inhibition) and non-dopaminergic (modulation of the abnormal glutamate release). The efficacy of safinamide in early and advanced parkinsonian patients has been investigated in double blind, placebo controlled randomized clinical trials. In early PD patients^16^, safinamide 100 mg/d induced a significant improvement in the UPDRS part III score versus placebo (−6.0 points versus −3.6 (*p* = 0.0419). This positive trend was confirmed in the following double-blind, placebo-controlled extension study^17^. In contrast, when added to DAs at 200 mg a day, safinamide failed to provide a significant improvement in motor functions in early PD patients.

In advanced patients, safinamide was associated to a significant improvement of motor symptoms, evaluated through the UPDRS-III (motor score) administered during the ON period, in the three pivotal trials performed. In the 016 Study^18^, the UPDRS-III significantly improved, both in the safinamide 50 mg and 100 mg groups compared to placebo (LS mean changes for the 50 mg/day: −1.8 [95% CI, −3.3 to −0.4; *p* = 0.0138]; for the 100 mg/day: −2.6 in hours [95% CI, −4.1 to −1.1; *p* = 0.00060]). These results were confirmed and maintained throughout the double-blind, placebo-controlled, parallel group, extension study^19^. In the SETTLE trial^20^, when evaluated in the on phase, the mean UPDRS-III score was found to improve significantly in the safinamide 100 mg group compared to placebo: –3.43 (7.72) vs –1.83 (8.23), (LS mean difference, –1.82; 95% CI, –3.01 to –0.62; *p* = 0.003).

The improvement reported in the UPDRS was clearer in early patients but was less evident in fluctuators when UPDRS was administered during ON and was not the primary endpoint. In early PD patients, the improvement observed with safinamide at 200 mg daily failed to reach statistical significance, probably in relation to the higher discontinuations rate registered in the group treated at this dosage, which may have masked the clinical benefit.

Regarding the efficacy on motor fluctuations, a high level of evidence supports the consensus obtained in this Delphi panel on motor fluctuations. Safinamide has been demonstrated to significantly increase on time without dyskinesia and with non-troublesome dyskinesia compared with placebo, in both the 016 Study (least squares mean change versus placebo for safinamide 50 mg/day 0.51 h; 95% CI, 0.07–0.94; *p* = 0.0223 and for 100 mg/day 0.55 h; 95% CI, 0.12–0.99; *p* = 0.0130) and the 018 Study (LS increase from baseline: 1.01 h for the safinamide 50 mg/d group, 95% CI, 0.23, 1.11; *p* = 0.0031 and 1.18 h for the 100 mg/d group, 95% CI; 0.39, 1.27; *p* = 0.0002)^18,19^. A parallel, significant decrease in off time was also noted (−0.6, *p* = 0.0043 and −0.6, *p* = 0.0034) in the 016 trial. Moreover, a significant increase in on time without troublesome dyskinesia (+1.42, least-squares mean difference, 0.96 h; 95% CI, 0.56−1.37 h; *p* < 0.001) was reported in the SETTLE Study^20^. The efficacy of safinamide on motor fluctuations and motor symptoms was shown in an uncontrolled study on 91 patients, which reported a significant reduction in daily off time from baseline (median 60 min vs 30, *p* = 0.0001), and in on time with dyskinesia; a significant improvement from baseline was also found in UPDRS part III and UDysRS item 9^21^. The recently published results of the SYNAPSES trial are in line with the literature, showing a progressive improvement of wearing off and early morning off over the 12 months study, a reduction in the percentage of fluctuating patients from 73.5% to 67.4%^22^ Two retrospective observational studies also highlighted the beneficial effect of this drug on fluctuations measured by the WOQ 19^23^ and CGI^24^.

The conflicting results obtained in this Delphi panel on dyskinesia reflect the uncertainty that emerged from clinical studies, where dyskinesia was the most frequently reported AE^18,20^. Indeed, in the 018 Study^19^ the primary endpoint, i.e., mean change from baseline (at Study 016 start^18^) to endpoint of the total score of the Dyskinesia Rating Scale (DRS) during on time, was not met. Nevertheless, a subsequent ad hoc analysis performed on moderately-to-severely dyskinetic patients who entered Study 016 (*n* = 242, 36%) showed a statistically significant reduction in the mean DRS total scores (*p* = 0.0317) and the same trend was observed in a post hoc analysis of the 018 study^25^, where safinamide, only when administered at 100 mg day, was found to improved DRS scores, regardless of LD changes. Moreover, in a retrospective study published recently, the introduction of safinamide was not associated with an improvement in the severity of dyskinesia, which remained unchanged in 85.4% of the enrolled population^24^. A potential anti-dyskinetic effect of safinamide may be related to a reduction in the excitatory overdrive of the direct pathway^26^. This characteristic distinguishes safinamide from amantadine, which is an anti-dyskinetic drug that directly inhibits glutamatergic receptors, but it is characterized by a somewhat unfavorable safety profile. Furthermore, a study to solely explore the effect of safinamide on dyskinesia has not been conducted yet and therefore it is difficult to infer a definite conclusion. The conflicting results on this topic may be also interpreted as an indication of the different therapeutic strategies adopted by neurologists when introducing safinamide in a therapeutic schedule, where the simultaneous adjustment of the concomitant dopaminergic drugs may have an important role in the severity of dyskinesia observed.

With respect to the glutamatergic system, all the panelists acknowledged that this plays an important role in PD aetiopathogenesis as well as in the emergence of its complications and that the anti-glutamatergic properties of safinamide may potentially explain an observed beneficial effect on dyskinesia and NMS. A dysfunction of both dopaminergic and non-dopaminergic pathways is known to contribute to development of NMS, thus drugs that interact with several neurotransmission systems may be helpful in alleviating these dysfunctions.

It has been suggested that the anti-glutamatergic properties of safinamide may be particularly beneficial in addressing motor complications and some specific NMS. This is a speculative comment but can be supported by animal models; there are data indicating the role of glutamatergic neurotransmission in modulating pain in normal and neuropathic rats^27,28^. Preclinical data suggest that the brain glutamate system may be involved in depression related behavior like anhedonia^29^. In humans with depression, neuroimaging studies found increased levels of glutamate in the putamen, highlighting a potential role of the basal ganglia in the neurophysiology of depression^30^. Glutamate was also hypothesized to influence the sleep-wake rhythm through the release of glutamate from the supra-mammillary region, which was associated to a sustained behavioral and EEG arousal response^31^.

A general consensus was reached on the efficacy of safinamide on pain, mood and sleep, based on literature findings. The possible effect of safinamide on NMS needs to be further explored in dedicated trials, since evidence currently available is limited^32^. Treatment with Safinamide was related to a long-lasting, significant reduction of pain-related sub-items of quality of life questionnaire (PDQ-39). Moreover, a reduction of concomitant pain killer treatment in fluctuating PD patients in a post-hoc analysis of the 018 study^33^ and in a pooled analysis of the 016 and SETTLE studies was observed^34^. A beneficial effect on pain was also shown in a small prospective study where safinamide was used to address pain related to motor fluctuations^35^. In a small retrospective analysis performed in fluctuating PD patients with sleep disruption, a significantly greater improvement of both, nocturnal sleep and diurnal sleepiness, was highlighted in the group of subjects treated with safinamide compared to those on rasagiline^36^.

Safinamide was found to significantly improve emotional well-being and depression for up to two years in a post hoc, pooled analysis of the 016 and 018 Studies^37^, even though in the original studies the improvement in the GRID Hamilton Rating Scale for Depression failed to reach statistical significance compared to placebo. In another retrospective study, safinamide was shown to significantly improve depressive symptoms in PD patient after just one month of treatment and to be well tolerated when co-administered with antidepressants^38^.

A beneficial effect on pain was shown in a retrospective survey and in a small prospective study^35^. In another retrospective study safinamide was shown to be useful for treating depressive symptoms in PD patients and safe when administered with concomitant antidepressant^38^. The positive effect on chronic pain and mood may be also related to the ability of safinamide to inhibit sodium channel^39,40^.

No agreement was obtained regarding domains like cognition, bladder, and sexual dysfunctions, highlighting the paucity of supporting evidence in these areas. Regarding cognition, in studies performed to explore the tolerability of safinamide in elderly and in general population, patients enrolled had no cognitive impairment (MMSE ≤ 23) or previous history of hallucinations;^24^ when patients with cognitive impairment were included, the number of withdrawals due to the occurrence of confusion was higher among patients with more severe cognitive impairment^24^. The lack of agreement on hallucinations reflects what emerged from post-marketing studies and in clinical practice where the use of safinamide was found to potentially increase the risk of hallucinations, most likely related to the higher dopaminergic load induced by the drug^23,24^.

The consensus on a likely lack of positive effects of safinamide on orthostatic hypotension was expected**:** even though the use of safinamide has been associated to a slight, non-significant raise in blood pressure values in pivotal trials^18,19^, the enhancements of the vasodilating action of dopaminergic drugs may explain the lack of tolerability observed in patients with OH. It has to be noted though that in a post-marketing study performed to evaluate the safety of the immediate switch from rasagiline to safinamide, blood pressure variability in systolic and diastolic values was unchanged between baseline and end of study 24-hour ambulatory blood pressure monitoring recordings^41^.

The beneficial effect of safinamide on QoL is supported by a high level of evidence. All studies that included QoL as an outcome parameter have demonstrated the efficacy of safinamide, when use at 100 mg, in benefiting both short-term and long-term quality-of-life outcomes in advanced PD patients measured by the PDQ-39 and the EQ5D questionnaires^18–20,42,43^.

The safety of safinamide was acknowledged by all panelists, in agreement with what had emerged from clinical trials in early^16,17^ and advanced PD patients^18–20^, where the majority of TEAE were mild to moderate in intensity and the most serious adverse events reported were judged as not related to the investigational drug. A large multinational, multicenter, retrospective, prospective 12-month observational cohort study evaluated the safety of safinamide in a real-life population of PD patients^22^ and showed a 30% lower rate of AEs compared to pivotal trials performed^18–20^. Subgroup analyses were performed for the following categories: age>75 years old, presence of relevant comorbidities and psychiatric conditions. Among these categories, SAEs were more frequent in patients aged >75 and AEs/SAEs were more frequent in those with relevant comorbidities. There were no cases of serotoninergic syndrome in participants treated with SSRI/SNRI or tricyclics, nor cases of impulse control disorders or sleep deterioration in patients taking concomitant dopamine-agonists. Dyskinesia was confirmed to be the most frequently reported AE, although observed at lower rates compared to the published RCTs (13.7% vs 18.3% for the 016 Study and 14.6% for the SETTLE)^18,20^. The SYNAPSES study^22^, conducted in a real word setting in six European countries, confirmed the safety and tolerability of safinamide, as adjunct therapy, in fluctuating patients and in special groups of subjects such as those suffering from neuropsychiatric symptoms (depression, bipolar disorders, psychosis and apathy).

The reversibility of the MAO-B inhibition of safinamide represents a clear advantage compared to the other MAO-B inhibitors when treating advanced PD patients with concomitant morbidities and high concomitant medications load, due to the reduced risk of drug interactions. Studies in healthy volunteers^7,8^ and patients^44^ have also confirmed that safinamide is not associated with any change of tyramine potentiation, with no risk of serotonin syndrome or hypertensive crisis.

Safinamide is a well-tolerated and easy to use drug thanks to its once-a-day administration. In fluctuating PD patients, this compound reduces OFF time without increasing troublesome dyskinesia. Its indirect anti-glutamatergic properties represent a novelty among the drugs for PD; its ability to reduce the hyperactivity of the direct pathway may potentially have long-term positive effect on the natural course of motor complications. Possibly due to its dual mechanism of action, it can be particularly useful in addressing NMS, whose treatment represents one of the current unmet needs of PD. Indeed, the ability to ameliorate NMS may be the most promising and distinctive feature of this compound: its potential efficacy in controlling pain, mood and sleep abnormalities may open the door to new therapeutics perspective if confirmed in randomized clinical trials.

The Delphi method requires participants to respond considering their personal experience/judgment on the proposed statements: the conclusions provided in this report are based therefore on the opinions of a panel of European movement disorder specialists, opinions maturated through a vast experience with the use of safinamide. The considerations highlighted in the paper may serve as a general reference for a more rational approach when prescribing safinamide, allowing movement disorders neurologists to further deepen, and not limit, their personal experience with this compound.

The consensus reached in this Delphi study provides an overview of how European movement disorders specialists view the role of safinamide in their clinical practice. The large agreement obtained reflects how specialists are mindful of the results of RCTs published and how these results guide their therapeutic perspective. The outcome almost perfectly reflects findings that have emerged in the literature and provides an interesting snapshot of the issues not yet addressed by the existing studies. Indeed, data on dyskinesia and NMS such as pain and mood are still limited, and more studies are needed to draw definite conclusion. However, clinical experience to date and the results of this Delphi process emphasizes that the data from controlled studies translate into a valuable clinical role of safinamide, which may be due to its dual mechanism of action, and this is true even at an early stage of motor fluctuations.

## Methods

### Study design and methods

The Delphi method is a survey technique that uses responses to a standardized questionnaire developed by a panel of experts to facilitate the convergence of opinions or the achievement of a common opinion in areas where scientific evidence is scarce or needed^45–47^. The Delphi method involves the repeated administration of questionnaires, where each statement can be evaluated through a five-point Likert scale, with a score from 1 to 5 (1, extremely disagree; 2, disagree; 3, agree; 4, mostly agree; and 5, extremely agree). Results are expressed as a percentage of respondents who scored each item as 1 or 2 (disagreement) or as 3, 4, or 5 (agreement). A positive consensus is reached if the percentage of agreement is greater than 66%. No consensus is reached, when the sum of the responses for a negative consensus (1 and 2) or a positive consensus (3, 4, and 5) is <66%.

### Delphi process

Eight European countries were involved in the project which took place between October 2020 and January 2021. The survey was developed by a board of ten movement disorders specialists, who represent expert in the treatment of PD in their respective countries. After reviewing the published literature on safinamide, the Board met to discuss the main areas of interest and identified six major topics: the role of glutamate in D, introduction to fluctuations, efficacy of safinamide on motor symptoms, motor complications and NMS, Quality of Life, safety of safinamide and Target Population. Each topic was subdivided in a variable number of statements corresponding to items where greater need of clarification and debate existed. The Board was also asked to identify 2 colleagues for each board member who served as external impartial validators and judged clarity and readability of the statements. The survey was then distributed via an online platform to 119 panelists who met the following profile: movement disorders specialists with at least five years’ experience and using safinamide in their clinical practice. The vote was anonymous, and no compensation was given to any of the identified voters.

The study does not report on, or involve the use of any animal or human data or tissue, and does not contain data from any individual person, so there was no need for ethics approval nor prior approval of the study protocol. All experts involved in the Delphi survey were informed of the study’s objectives and the possibility of publishing the results in a peer-reviewed article. The participation was voluntary. They expressed their consent to participate in the survey after logging into the secure online survey platform via credentials, by actively clicking on the appropriate box.

### Members of the validation panel

Valentina Leta (UK), Jasmin Rahimi (Austria), Katarina Rukavina (UK), Daniele Urso (UK)

### Panelists

Araceli Alonso (Spain), Lucia Batzu (UK), Anna Rita Bentivoglio (Italy), Filip Bergquist (Sweden), Marta Blázquez Estrada (Spain), Alexandra Boogers (Belgium), Christof Brücke Brücke (Austria), Nuria Caballol Pons (Spain), Paolo Calabresi (Italy), Camille Carroll (Uk), Buhmann Carsten (Germany), Roberto Ceravolo (Italy), Roberto Cilia (Italy), Carlo Colosimo (Italy), Radu Constantinescu (Sweden), Pietro Cortelli (Italy), David Crosiers (Belgium), Beatriz De La Casa Fages (Spain), Gino De la Meilleure (Belgium), Mieke De Weweire (Belgium), Woitalla Dirk (Germany), Atbin Djamshidian (Austria), Laurens Dobbels (Belgium), Christian Dresel (Germany), Georg Ebersbach (Germany), Karla Eggert (Germany), Reinhard Ehret (Germany), Roberto Eleopra (Italy), Roberto Erro (Italy), Francisco Escamilla Sevilla (Spain), Giovanni Fabbrini (Italy), Thomas Foki (Austria), Pieret Françoise (Belgium), Rocío García-Ramos (Spain), Ransmayr Gerhard (Austria), Juan Carlos Gomez Esteban (Spain), Ayoze Nauzet González Hernández (Spain), Victoria Haunton (UK), Jorrit Hoff (Netherlands), Jagdish Sharma Jagdish (UK), Kassubek Jan (Germany), Maes Jen (Belgium), Leenders Jo (Belgium), Anders Johansson (Sweden), Winkler Juergen (Germany), Vinod Metta (UK), Ines Legarda (Spain), Johan Lökk (Sweden), Leonardo Lopiano (Italy), Michael Lorrain (Germany), Rosario Luquin (Spain), Martina Müngersdorf (Germany), Juan Carlos Martínez Castrillo (Spain), Pablo Mir (Spain), Thomas Müller (Germany), Nina De Klippel (Belgium), Bruggemann Norbert (Germany), Dag Nyholm (Sweden), Sven Pålhagen (Sweden), Santens Patrick (Belgium), Manuela Pilleri (Italy), Andrea Pilotto (Italy), Monika Pötter-Nerger (Germany), Rocco Quatrale (Italy), Silvia Ramat (Italy), Jason Raw (UK), Heinz Reichmann (Germany), Mario Giorgio Rizzone (Italy), Pilar Sanchez (Spain), Diego Santos García (Spain), Klaus Seppi (Austria), Ángel Sesar (Spain), Nishantha Silva (UK), Monty Silverdale (UK), Örjan Skogar (Sweden), Antonio Suppa (Italy), Per Svenningsson (Sweden), JP ter Bruggen (Netherlands), Alessandro Tessitore (Italy), Warnrecke Tobias (Germany), Lars Toenges (Germany), Volker Tomantschger (Austria), Thomas Vaterrodt (Germany), Pieter Viaene (Belgium), Dieter Volc (Austria), Hofmann W. E. (Germany), Karoline Wenzel (Austria), Christian Winkler (Germany), Mario Zappia (Italy), Anna Lena Zecchinelli (Italy).

### Reporting summary

Further information on research design is available in the Nature Research Reporting Summary linked to this article.

## Supplementary information

REPORTING SUMMARY

## Data Availability

The data that support the findings of this study are available from the corresponding author upon reasonable request.
